# Factors associated with pressure injuries in bedridden older adults in Primary Health Care

**DOI:** 10.1590/0034-7167-2025-0163

**Published:** 2026-10-02

**Authors:** Jessica Fernandes dos Santos, Ana Beatriz Marques Fonseca, Leticia Dantas Teixeira, Jéssica Naiara de Medeiros Araújo, Maria Isabel da Conceição Dias Fernandes, Ana Luisa Brandão de Carvalho Lira, Jéssica Dantas de Sá Tinôco

**Affiliations:** I Universidade do Estado do Rio Grande do Norte. Caicó, Rio Grande do Norte, Brazil; II Universidade Federal do Rio Grande do Norte. Natal, Rio Grande do Norte, Brazil; III Universidade Federal do Ceará. Natal, Ceará, Brazil

**Keywords:** Pressure Ulcer, Bedridden Persons, Aged, Homebound Persons, Risk Factors., Úlcera por Presión, Diagnóstico de Enfermería, Atención Primaria de Salud, Personas Encamadas, Anciano.

## Abstract

**Objectives:**

to identify factors associated with the occurrence of pressure injuries (PI) in bedridden older adults treated in Primary Health Care (PHC).

**Methods:**

this cross-sectional study was conducted with 63 bedridden older adults. Validated clinical assessment instruments were applied, including physical skin examination, the Braden Scale, the Katz test, and indicators of nursing diagnoses from NANDA-International.

**Results:**

although all presented a risk for pressure injuries, a confirmed diagnosis occurred in 25.4%. The variables most strongly associated were non-intact skin (p<0.001), age spots (p<0.001), erythema (p=0.002), as well as medication use (p=0.024), decreased turgor (p=0.035), excessive moisture (p=0.032), previous history of pressure injuries (p=0.015), and altered sensitivity (p=0.021).

**Conclusions:**

early recognition of these factors can guide individualized care plans and standardized protocols in primary health care, strengthening the prevention of an avoidable condition.

## RESUMEN

**Objetivos:**

identificar los factores asociados con la aparición de úlceras por presión (UPP) en ancianos encamados atendidos en Atención Primaria de Salud (APS).

**Métodos:**

se realizó un estudio transversal con 63 ancianos encamados. Se aplicaron instrumentos de evaluación clínica validados, incluyendo la exploración física de la piel, la Escala de Braden, el test de Katz e indicadores de diagnósticos enfermeros de la NANDA-Internacional.

**Resultados:**

aunque todos los pacientes presentaron riesgo de UPP, el diagnóstico se confirmó en el 25,4%. Las variables con mayor asociación fueron piel no intacta (p<0,001), manchas seniles (p<0,001), eritema (p=0,002), así como el uso de medicamentos (p=0,024), disminución de la turgencia (p=0,035), humedad excesiva (p=0,032), antecedentes de UPP (p=0,015) y sensibilidad alterada (p=0,021).

**Conclusiones:**

el reconocimiento temprano de estos factores puede orientar la planificación individualizada de la atención y los protocolos estandarizados en atención primaria, fortaleciendo así la prevención de una afección evitable.

## INTRODUCTION

The bedridden older population is predisposed to developing skin injuries and delayed healing phases due to vascular changes, capillary fragility, nutritional deficiencies, and dry skin. Furthermore, excessive sun exposure and thinning at the dermis and epidermis junction, which causes wrinkles, depressions, and overlapping skin folds, are also significant factors^(1)^. Given these conditions and under the influence of extrinsic factors such as the external environment, friction, shear, and moisture, bedridden older adults are more vulnerable to developing localized skin injuries, especially pressure injuries (PIs)^(2)^.

PIs are defined as damage to the skin and soft tissues, usually over bony prominences or associated with the use of medical devices, resulting from intense and/or prolonged shear-related pressure. They can be classified into stages 1 to 4 or as non-stageable, representing, in ascending order, the extent of damage to the skin^(2)^. This is a complex problem that causes distress to bedridden older adults, their families, and healthcare professionals, with a significant presence in hospital settings and, increasingly, in home care, where Primary Health Care (PHC) acts as the main point of care. In addition to affecting quality of life, PIs generate significant costs for healthcare systems^(3)^. Despite scientific advances, it is still considered a challenge for bedridden older adults, their families, professionals, and healthcare services^(4)^.

Bedridden older adults with reduced mobility receiving home care have a high risk of developing PIs. The literature indicates a prevalence of up to 75% in this population^(5)^, a worrying figure, since, since 1980, there has been evidence that most cases could be avoided^(6,7)^. In this sense, PI prevention within PHC is strategic, with Family Health Strategy (FHS) teams playing a central role in clinical surveillance, caregiver education, and implementation of preventive actions, also serving as an indicator of quality of care^(4,7)^. Therefore, identifying factors associated with increased vulnerability in bedridden older adults should be prioritized, guiding preventive measures and strengthening care.

The systematic assessment of these factors constitutes a key prevention strategy, especially when integrated into the Nursing Process (NP) and the longitudinal care routines of the FHS. To this end, a scientific foundation is indispensable to support standardized protocols and improve quality of care^(8)^. The implementation of the NP in PHC, as a socio-environmental space for nursing care^(9)^, allows the identification of nursing diagnoses (NDs) related to the occurrence and risk of PIs. The NDs described in NANDA-International (NANDA-I)^(10)^ Risk for Adult Pressure Injury (RAPI) and Adult Pressure Injury (API) present clinical indicators useful for the precise identification of the injury or vulnerability to its development.

In this context, understanding the predisposing factors for PIs is fundamental for clinical assessment and the adoption of preventive measures. Furthermore, it is essential that nursing professionals know the main causes of the problem in bedridden older adults in PHC, in order to develop effective care plans^(11)^. Understanding these associations can guide evidence-based practices, strengthen public policies for healthy aging, and respond to the growing demand for qualified home care. It is hoped, therefore, that this study will contribute to strengthening clinical practice in PHC, guided by evidence and focused on the prevention of avoidable complications.

## OBJECTIVES

To identify factors associated with the development of PIs in bedridden older adults receiving care in PHC.

## METHODS

### Ethical aspects

The project received ethical approval. Informed Consent Form was obtained from all individuals involved in the study and signed in writing.

### Study design, period, and site

This is a cross-sectional study, guided by the STrengthening the Reporting of OBservational studies in Epidemiology tool, conducted in the municipality of Caicó, Rio Grande do Norte. Data collection took place in households between March and July 2023.

### Population and sample

The population consisted of bedridden older adults. A formula designed for finite populations was used to calculate the sample size, considering the confidence coefficient, sampling error, and population size^(12)^, namely: n= Z^2^∞.P.Q.N/e^2^(N-1)+Z^2^.∞P.Q. The letters in the formula mean the following: n = sample size; Z∞ = confidence level of the study; e = absolute sampling error; N = population size; Q = complement of prevalence (100-P); P = prevalence. Thus, the parameters used in the sample size calculation were a 95% confidence level (Zα = 1.96) and a 5%sampling error.

The Brazilian National Household Sample Survey (continuous), in 2017, identified that 14.6% of the Brazilian population was 60 years of age or older. Among these, 4.9% were bedridden^(13)^. Considering that the municipality of Caicó has 68,343 inhabitants^(14)^, an estimated population of 9,978 older adults was found, of which 489 were bedridden. Thus, applying the formula for sample size calculation in finite populations, the final size of 63 bedridden older adults was obtained.

The sample was obtained through multi-stage cluster sampling, a strategy chosen due to the territorial organization of FHS teams in the municipality, which favors the representativeness of the different coverage areas. Thus, the quantity of 63 bedridden older adults was distributed among 21 FHS teams. Three bedridden older adults were randomly selected per FHS team. In the second stage, a survey of the micro-areas where bedridden older adults resided was conducted, and among these, three micro-areas from each FHS team were randomly selected. In the third stage, one bedridden older adult per micro-area, selected in the second stage, was randomly selected.

### Inclusion and exclusion criteria

Inclusion criteria comprised being ≥ 60 years of age, being bedridden at home during the data collection period, having preserved cognitive capacity to answer the questions, as clinically assessed by the researchers during the home visit, or, in the absence of this condition, having a responsible person/caregiver who could answer the form, and being registered at a Basic Health Unit in the city of Caicó, Rio Grande do Norte. Exclusion criteria comprised residing in the rural area of Caicó, Rio Grande do Norte, and being hospitalized during the data collection period.

### Study protocol

Data collection was carried out by three researchers previously trained in the application of the instruments and standardization of the clinical assessment. The training included a theoretical review of the instruments, practical simulations, and case discussions, ensuring uniformity in the identification of variables. Home visits were accompanied by community health workers, lasting an average of 75 minutes each, and included an interview with the bedridden older adult or caregiver and a focused physical examination.

The data collection instrument was developed from validated instruments and indicators from the literature, structured in three chunks: (a) sociodemographic and clinical characterization (previous illnesses, length and reason for bed rest, medications in use); (b) functional and risk assessment, using the Katz Index (independence in basic activities of daily living) and the Braden Scale (risk assessment for developing PIs); and (c) skin physical examination, comprising items from the NANDA-I 2024-2026 taxonomy for the RAPI and API NDs, which include clinical indicators such as non-intact skin, erythema, decreased turgor, age spots, and excessive moisture.

Variables were obtained from the direct application of these instruments during the home visit, and recorded on a structured form containing closed-ended questions and clinical observation items. All instruments used have prior validity in the literature. In the case of the NANDA-I NDs, validity stems from international standardization, while the Katz Index and the Braden Scale have consolidated evidence of clinical use.

### Analysis of results and statistics

Diagnostic inference was performed by two researchers based on the occurrence of clinical indicators of NDs in the sample. When there was no consensus, a third researcher was called upon to make a final decision.

Data were organized using Microsoft Office Excel^®^, and descriptive analysis of variables was performed using the Statistical Package for the Social Sciences version 25.0. Descriptive statistics were performed using relative and absolute frequencies, as well as measures of central tendency and dispersion. The Kolmogorov-Smirnov test was also used to verify data normality.

Inferential measures were obtained using Pearson’s chi-square test, and Fisher’s exact test was used when the expected frequencies were less than five. In all tests, a significance level of 5% (p≤0.05) was adopted.

## RESULTS

The research sample consisted of 63 bedridden older adults attended by PHC. Furthermore, 57.1% were female, with a mean age of 83.32 years (±9.381), a minimum age of 60 years, and a maximum age of 98 years. Regarding age range, the following age groups were identified: 60 to 70 years (9.5%), 71 to 80 years (31.7%), 81 to 90 years (33.3%), and over 90 years (25.4%).

Concerning marital status, 49.2% were widowed. Concerning race, the majority were white (58.7%), and the minority were black (4.8%). In terms of education level, 58.8% were illiterate. In relation to family income, 77.7% had an income of up to two minimum wages. And concerning the number of dependents, three was the median number. Furthermore, 63.5% lived with their children, and 84.1% had a family member as their caregiver.

As for clinical data, most older adults have pre-existing conditions such as hypertension (47.6%) and central nervous system diseases (34.9%), while 11.1% have no diagnosis. Regarding medications, the use of antihypertensives (38.1%), antipsychotics (23.8%), and acetylcholinesterase inhibitors (20.6%) stands out.

Regarding the length of time spent in bed, 41.3% had been bedridden for at least 24 months, with a median of 36.6 months, a minimum of four months, and a maximum of 912 months. Among the reasons that led older adults to become bedridden, the most prominent were accidents and fractures (22.2%), sequels of stroke (19%), Alzheimer’s disease (17.4%), and fatigue and muscle weakness (15.9%).

The Katz test results, which assesses the level of dependence in performing activities of daily living, showed that 60% of bedridden older adults are dependent for all activities, and 20% are dependent for five activities.

The ND RAPI was identified in 100% of those surveyed. The most relevant (external) risk factors were surface friction (100%), pressure on bony prominences (100%), shear forces (74.6%), use of sheets with insufficient absorption and elimination properties (68.3%), continuous mechanical load (71.4%), inadequate access to appropriate equipment (65.1%), and inadequate caregiver knowledge about PI prevention strategies (63.5%). As for risk factors (internal), decreased physical activity (100%), inadequate knowledge about PI prevention strategies (96.8%), decreased physical mobility (93.7%), inadequate adherence to the PI prevention plan (60.3%), and dehydration (49.2%) stand out.

In relation to the Braden Scale results, 68.2% of older adults presented a low to moderate risk. Of the at-risk population, 100% were older adults with physical disabilities; 96.8% were individuals receiving home care; and 31.7% had a history of PIs. Regarding associated conditions, 38.1% had decreased tissue perfusion; 34.9% had chronic neurological conditions; 28.6% had diseases of the central nervous system; and 27% had impaired circulation.

The ND API defining characteristics indicated a prevalence of 25.4% of the diagnosis in the population of bedridden older adults assisted by PHC. Moreover, research participants presented the following characteristics: purple localized area of discolored intact skin (17.5%); erythema (14.3%); partial thickness loss of dermis (14.3%); pain at pressure points (11.1%); full thickness tissue loss (6.3%); full thickness tissue loss with exposed muscle (1.6%); full thickness tissue loss with exposed bone (1.6%); ulcer is covered by eschar (1.6%); and ulcer is covered by slough (1.6%).

In light of the above, it is observed that, of the bedridden older adults who presented with the ND API, 17.5% developed only one injury, 4.8% two injuries, 1.6% five injuries, and 1.6% seven injuries. The classifications of these injuries varied between stage 1, stage 2, stage 3, deep tissue, and unstageable.

Older adults with and without PIs were correlated with the following variables: sociodemographic characteristics (age, sex, race, marital status, education level, individual income source, family income, number of dependents, housing arrangement, and presence of a caregiver); general clinical data (previous illnesses, medication in use, length and reason for bed rest); clinical skin characteristics (integrity, color, moisture, texture, turgor, thickness, sensitivity, temperature, mobility, and edema); and clinical indicators of NDs RAPI and API (risk factors, at-risk population, and associated conditions).

Table 1 presents sociodemographic and clinical variables, as well as the indicators of NDs that showed a statistically significant association with the occurrence of PIs in bedridden older adults.

**Table 1:** Significant statistical association between bedridden older adults who presented with pressure injuries compared to those who did not, based on sociodemographic and clinical variables and indicators of nursing diagnoses Risk of Pressure Injury in Adults and Pressure Injury in Adults, Caicó, Rio Grande do Norte, Brazil, 2023

Variables	*p* value
Use of medications	0.024^†^
Non-intact skin	<0.001 ^*^
Age spots	<0.001 ^*^
Painful sensitivity	0.021 ^*^
Tactile sensitivity	0.021 ^*^
Rough skin	0.056^†^
Decreased turgor	0.035 ^*^
Erythema	0.002 ^*^
Excessive moisture	0.032 ^*^
Use of bed sheets with insufficient moisture absorption and wicking properties	0.056 ^*^
Individuals with a history of pressure injury	0.015 ^*^

* *Pearson’s chi-square test; †Fisher’s exact test.*

The association between bedridden older adults with PIs compared to those without, starting at a significance level of 5%, showed statistical relevance among 11 variables, which were interpreted as factors associated with the occurrence of PIs. These variables were: non-intact skin; erythema; age spots; medication use; rough skin; decreased turgor; excessive moisture; use of sheets with insufficient moisture absorption and wicking properties; individuals with a history of PIs; pain sensitivity; and tactile sensitivity.

## DISCUSSION

PI prevention and early management should be carried out in an integrated manner, considering clinical, educational, and managerial aspects^(6)^. This study analyzed the sociodemographic and clinical profile, the occurrence of NDs RAPI and API, as well as factors associated with the development of PIs in bedridden older adults receiving PHC. This approach reinforces the clinical use of NDs as a guiding tool for decision-making and management of home care.

The identified sociodemographic profile showed a predominance of females, white race, widowed marital status, and low education level, with a mean age of 83.32 years (±9.381). These findings are similar to other research in home care^(15)^, confirming the aging population, particularly in the 80 years and older age group. Furthermore, the majority have low family incomes, revealing social vulnerability in the face of caregiving demands^(15)^.

In clinical settings, the following stood out: arterial hypertension, nervous system diseases, and frequent use of antihypertensive and antipsychotic drugs, in addition to causes of bedriddenness such as accidents, fractures, sequels of stroke, and Alzheimer’s disease, in line with the literature^(16)^. The Katz test confirmed high functional dependence, highlighting the need for caregiver support strategies, such as educational workshops, preventive home visits, and the use of checklists for monitoring^(17)^. Such health promotion practices are fundamental to reducing risks and strengthening a preventive culture at home.

Skin changes related to aging, such as decreased elasticity, increased mobility, thin texture, and the presence of age spots, increase the vulnerability of bedridden older adults to PIs^(18,19)^. The ND RAPI was identified in 100% of the sample, reinforcing the prevalence of risk in this population^(20)^. Early identification by a nurse is essential to prevent complications, which highlights the importance of the NP^(8)^. These results are consistent with current public policies, such as the Brazilian National Health Policy for Older Adults and the Brazilian National Health Regulatory Agency (In Portuguese, *Agência Nacional de Vigilância Sanitária* - ANVISA) recommendations on patient safety^(7,21)^, reinforcing PI as a strategic indicator of quality of care and the need for alignment with national care protocols.

The prevalence of 25.4% of ND API found in this study is similar to that of other research, which indicates rates between 0.5% and 22.5%^(21-23)^. This data reinforces PIs as a serious public health problem in the home setting and a challenge for nursing clinical practice. The institutionalization of screening protocols, skin monitoring, and care flows can enhance prevention and improve the quality of care, as well as align with national policies for elder healthcare and patient safety.

Among the factors significantly associated with PIs, non-intact skin (p<0.001), age spots (p<0.001), and erythema (p=0.002) stood out; these alterations are directly observable in the clinical examination and constitute signs of high risk. Variables such as medication use (p=0.024), decreased turgor (p=0.035), excessive moisture (p=0.032), previous history of PIs (p=0.015), and alterations in pain and tactile sensitivity (p=0.021) also showed relevance, supporting findings from previous studies^(13,24-30)^. These results indicate critical points that should be prioritized in prevention protocols and systematic monitoring.

The use of continuous medications, such as antihypertensives, antipsychotics, and sedatives, has been associated with increased vulnerability, as they reduce tissue perfusion, impair the level of consciousness, and hinder mobility, making spontaneous changes in position more difficult^(18,25,31,32)^. Neurological diseases, such as stroke and Alzheimer’s, also interfere with sensory perception, increasing the risk of PIs^(26,27)^. Furthermore, a prior history of PIs increases the likelihood of recurrence, making prevention even more challenging^(33)^.

The findings of this study not only reveal clinical vulnerabilities but also provide practical perspectives for PHC, such as the implementation of skin screening protocols, strengthening the continuing training of nursing professionals, the use of caregiver support tools, and the incorporation of digital monitoring technologies. Future research should further analyze the magnitude of the associations found through longitudinal and multivariate designs, as well as assess the effectiveness of educational and interdisciplinary strategies in home settings.

### Study limitations

The methodological limitations of this study are its cross-sectional design and the lack of precise nutritional assessment, due to the impossibility of obtaining the Body Mass Index. Despite this, the results obtained provide concrete information for improving care, focusing on the development of care protocols that guide skin care for bedridden older adults. Future studies, with longitudinal designs and systematic nutritional assessment, may broaden the understanding of predictive factors for PIs, strengthening the scientific basis for preventive protocols.

Also noteworthy is the absence of analysis of association measures, such as the Odds Ratio and its Confidence Intervals, which could indicate the magnitude of the effect of the variables associated with PI. Thus, the findings indicate the existence of statistically significant associations, but do not allow us to determine the strength of these relationships. It is suggested that future research should conduct multivariate analyses to deepen the understanding of predisposing factors.

### Contributions to nursing, health, or public policy

The study’s contributions, based on the identification of factors associated with the occurrence of PIs, reinforce the need to strengthen PI prevention actions within PHC. FHS teams should play a leading role in the early identification of risk factors and in the implementation of educational and care strategies in home care. The NP and the qualified use of NDs, such as RAPI and API, can guide evidence-based practices, promote continuous monitoring of skin integrity, and support clinical decisions that prevent avoidable complications.

In this context, the creation of practical tools to support nursing practice is recommended, such as risk checklists, training workshops, and interdisciplinary protocols focused on PI prevention in PHC, based on the results of this study. Aligning these contributions with Brazilian National Health Policy for the Older Adult guidelines and ANVISA recommendations on patient safety reinforces the strategic role of PHC in preventing avoidable complications. Furthermore, health promotion practices, such as self-care education, caregiver training, and community campaigns, can extend preventive reach beyond the home. It is suggested that future research explore the effectiveness of interdisciplinary protocols and digital skin monitoring technologies in home settings, expanding the potential for practical application of the findings.

## CONCLUSIONS

Factors associated with pressure injury in bedridden older adults in PHC were identified as including non-intact skin, erythema, age spots, medication use, rough skin, decreased turgor, excessive moisture, use of sheets with insufficient absorption, previous history of pressure injury, and reduced pain and tactile sensitivity. These findings highlight clinical vulnerabilities that, if recognized early, can support strategic prevention interventions in the area.

The occurrence of NDS RAPI in 100% of the sample and API in 25.4% highlights the high exposure and real impact of the condition in this population. These results can support the development of municipal prevention protocols, integrating skin monitoring into home visits by the FHS and including personalized care plans, continuing education for nursing teams, caregiver training, systematic skin monitoring, early interventions, and environmental control strategies. The incorporation of digital tools to support clinical decision-making and educational applications aimed at caregivers represents a promising innovation to enhance the effectiveness of these strategies.

Hence, the study provides support for strengthening the central role of PHC professionals, especially nurses, in the early identification, prevention, and mitigation of PIs, using the NP as a guiding instrument. Longitudinal and multicenter studies are recommended to assess the progression of the identified factors, as well as intervention trials aimed at training caregivers and nursing teams. This is expected to contribute to strengthening care practices and preserving the skin integrity, dignity, and quality of life of older adults at home.

## Data Availability

The research data are available within the article.
