# Supplementary material for: Factors associated with pressure injuries in bedridden older adults in Primary Health Care
Source: Rev Bras Enferm. 2026 Oct 2;79:e20250163. doi: 10.1590/0034-7167-2025-0163 (PMC13631951; doi:10.1590/0034-7167-2025-0163)
Supplement: Translation

# Translation: Fatores associados à lesão por pressão em pessoas idosas acamadas na Atenção Primária à Saúde

**DOI:** 10.1590/0034-7167-2025-0163pt

**Authors:** Jessica Fernandes dos Santos, Ana Beatriz Marques Fonseca, Leticia Dantas Teixeira, Jéssica Naiara de Medeiros Araújo, Maria Isabel da Conceição Dias Fernandes, Ana Luisa Brandão de Carvalho Lira, Jéssica Dantas de Sá Tinôco

**Affiliations:** I Universidade do Estado do Rio Grande do Norte. Caicó, Rio Grande do Norte, Brasil; II Universidade Federal do Rio Grande do Norte. Natal, Rio Grande do Norte, Brasil; III Universidade Federal do Ceará. Natal, Ceará, Brasil

**Keywords:** Úlcera por Pressão, Diagnóstico de Enfermagem, Atenção Primária à, Saúde, Pessoas Acamadas, Idoso.

## Abstract

**Objetivos:**

identificar fatores associados à ocorrência de lesão por pressão (LPP) em pessoas idosas acamadas atendidas na Atenção Primária à Saúde (APS).

**Métodos:**

estudo transversal realizado com 63 pessoas idosas acamadas. Foram aplicados instrumentos validados de avaliação clínica, incluindo exame físico da pele, Escala de Braden, teste de Katz e indicadores dos diagnósticos de enfermagem da NANDA-Internacional.

**Resultados:**

embora todas apresentassem risco para LPP, o diagnóstico confirmado ocorreu em 25,4%. As variáveis mais fortemente associadas foram pele não íntegra (p<0,001), mancha senil (p<0,001), eritema (p=0,002), além de uso de medicamentos (p=0,024), turgor diminuído (p=0,035), umidade excessiva (p=0,032), histórico prévio de LPP (p=0,015) e alterações de sensibilidade (p=0,021).

**Conclusões:**

o reconhecimento precoce desses fatores pode orientar planos de cuidados individualizados e protocolos padronizados na APS, fortalecendo a prevenção de um agravo evitável.

## INTRODUÇÃO

A população de pessoas idosas acamadas está predisposta ao desenvolvimento de lesões na pele e ao retardo das fases de cicatrização em virtude de alterações vasculares, fragilidade capilar, deficiência nutricional e ressecamento da pele. Outrossim, destacam-se ainda a exposição excessiva ao sol e o adelgaçamento na junção da derme e epiderme, que provoca rugas, depressões e sobreposição das pregas cutâneas^(1)^. Diante dessas condições e sob influência de fatores extrínsecos, como meio externo, fricção, cisalhamento e umidade, a pessoa idosa acamada apresenta maior vulnerabilidade ao desenvolvimento de lesões localizadas na pele, principalmente a lesão por pressão (LPP)^(2)^.

A LPP é definida como dano na pele e em tecidos moles, geralmente em proeminência óssea ou associada ao uso de dispositivos médicos, resultante de pressão intensa e/ou prolongada relacionada ao cisalhamento. Pode ser classificada em estágios de 1 a 4 ou em não estadiável, representando, em ordem crescente, a extensão do dano ocasionado na pele^(2)^. Trata-se de problema complexo e causador de transtornos à pessoa idosa acamada, seus familiares e profissionais de saúde, com presença significativa nos ambientes hospitalares e, de forma crescente, no cuidado domiciliar, onde a Atenção Primária à Saúde (APS) atua como principal frente assistencial. Além de afetar a qualidade de vida, a LPP gera custos expressivos para os sistemas de saúde^(3)^. Apesar dos avanços científicos, ainda é considerada um desafio para a pessoa idosa acamada, familiares, profissionais e serviços de saúde^(4)^.

A pessoa idosa acamada com mobilidade reduzida em cuidado domiciliar apresenta risco elevado para o desenvolvimento de LPP. A literatura aponta prevalência de até 75% nessa população^(5)^, dado preocupante, uma vez que, desde 1980, existem evidências de que a maioria dos casos poderia ser evitada^(6,7)^. Nesse sentido, a prevenção da LPP no âmbito da APS é estratégica, com papel central das equipes da Estratégia Saúde da Família (ESF) na vigilância clínica, educação de cuidadores e implementação de ações preventivas, configurando-se também como indicador da qualidade da assistência^(4,7)^. Assim, a identificação dos fatores associados ao aumento da vulnerabilidade da pessoa idosa acamada deve ser priorizada, orientando medidas preventivas e o fortalecimento do cuidado.

A avaliação sistemática desses fatores constitui estratégia-chave de prevenção, sobretudo quando integrada ao Processo de Enfermagem (PE) e às rotinas de cuidado longitudinal da ESF. Para tanto, é indispensável fundamentação científica que subsidie protocolos padronizados e qualificação da assistência^(8)^. A implementação do PE na APS, enquanto espaço socioambiental do cuidado de enfermagem^(9)^, permite identificar diagnósticos de enfermagem (DEs) relativos à ocorrência e ao risco de LPP. Os DEs descritos na NANDA-Internacional (NANDA-I)^(10)^ Risco de Lesão por Pressão no Adulto (RLPPA) e Lesão por Pressão no Adulto (LPPA) apresentam indicadores clínicos úteis à identificação precisa da lesão ou da vulnerabilidade para seu desenvolvimento.

Nesse contexto, compreender os fatores predisponentes à LPP é fundamental para a avaliação clínica e adoção de medidas preventivas. Além disso, é essencial que o profissional de enfermagem conheça as principais causas do problema em pessoas idosas acamadas na APS, de modo a elaborar planos de cuidados eficazes^(11)^. A compreensão dessas associações pode orientar práticas baseadas em evidências, fortalecer políticas públicas de envelhecimento saudável e responder à crescente demanda por cuidado domiciliar qualificado. Espera-se, assim, que este estudo contribua para o fortalecimento da prática clínica na APS, orientada por evidências e voltada à prevenção de agravos evitáveis.

## OBJETIVOS

Identificar os fatores associados ao surgimento da LPP em pessoas idosas acamadas atendidas na APS

## MÉTODOS

### Aspectos éticos

O projeto teve aprovação ética. O Termo de Consentimento Livre e Esclarecido foi assinado por todos os indivíduos envolvidos no estudo por meio escrito.

### Desenho, período e local do estudo

Trata-se de estudo transversal, norteado pela ferramenta *STrengthening the Reporting of OBservational studies in Epidemiology*, realizado no município de Caicó, Rio Grande do Norte. A coleta de dados ocorreu no domicílio entre os meses de março e julho de 2023.

### População e amostra

A população foi composta por pessoas idosas acamadas. Para o cálculo da amostra, foi utilizada uma fórmula destinada a populações finitas, considerando o coeficiente de confiança, o erro amostral e o tamanho da população^(12)^, a saber: n= Z^2^∞.P.Q.N/e^2^(N-1)+Z^2^.∞P.Q. As letras da fórmula significam o seguinte: n = tamanho da amostra; Z∞ = nível de confiança do estudo; e = erro amostral absoluto; N = tamanho da população; Q = complementar da prevalência (100-P); P = prevalência. Desse modo, os parâmetros empregados no cálculo amostral foram nível de confiança de 95% (Zα = 1,96) e erro amostral de 5%.

A Pesquisa Nacional por Amostra de Domicílios (contínua), em 2017, identificou que 14,6% da população brasileira tinha idade igual ou superior a 60 anos. Entre esses, 4,9% encontravam-se em condição de acamamento^(13)^. Considerando que o município de Caicó possui 68.343 habitantes^(14)^, estimou-se uma população de 9.978 pessoas idosas, das quais 489 estavam acamadas. Assim, aplicando-se a fórmula para cálculo amostral em populações finitas, obteve-se o tamanho final de 63 pessoas idosas acamadas.

A amostra foi obtida por meio de amostragem por conglomerados em múltiplas etapas, estratégia escolhida devido à organização territorial das equipes da ESF no município, o que favorece a representatividade das diferentes áreas de cobertura. Assim, o quantitativo de 63 pessoas idosas acamadas foi distribuído para 21 equipes de ESF. Foram sorteadas três pessoas idosas acamadas por ESF. Na segunda etapa, realizou-se o levantamento das microáreas onde residiam pessoas idosas acamadas, e entre essas, foram sorteadas três microáreas de cada ESF. Na terceira etapa, foi sorteada uma pessoa idosa acamada por microárea, selecionada na segunda etapa.

### Critérios de inclusão e exclusão

Os critérios de inclusão utilizados foram ter idade ≥ 60 anos de idade, estar acamado no domicílio no período da coleta de dados, apresentar capacidade cognitiva preservada para responder às perguntas, avaliada clinicamente pelos pesquisadores durante a visita domiciliar, ou, na ausência dessa condição, dispor de um responsável/cuidador que respondesse ao formulário, e estar cadastrado em uma Unidade Básica de Saúde da cidade de Caicó, Rio Grande do Norte. Já os critérios de exclusão foram residir na zona rural de Caicó, Rio Grande do Norte e estar vivenciando internação hospitalar no período da coleta de dados.

### Protocolo do estudo

A coleta de dados foi realizada por três pesquisadoras previamente treinadas para aplicação dos instrumentos e padronização da avaliação clínica. O treinamento incluiu revisão teórica dos instrumentos, simulações práticas e discussão de casos, assegurando uniformidade na identificação das variáveis. As visitas domiciliares foram acompanhadas por agentes comunitários de saúde, com duração média de 75 minutos cada, contemplando entrevista com a pessoa idosa acamada ou cuidador e exame físico direcionado.

O instrumento de coleta de dados foi elaborado a partir de instrumentos validados e indicadores da literatura, estruturado em três blocos: (a) caracterização sociodemográfica e clínica (doenças prévias, tempo e motivo de acamamento, medicações em uso); (b) avaliação funcional e de risco, por meio do Índice de Katz (independência em atividades básicas de vida diária) e da Escala de Braden (avaliação de risco para desenvolvimento de LPP); e (c) exame físico da pele, composto por itens da taxonomia NANDA-I 2024-2026 para os DEs RLPPA e LPPA, que contemplam indicadores clínicos como pele não íntegra, eritema, turgor diminuído, mancha senil e umidade excessiva.

As variáveis foram obtidas a partir da aplicação direta desses instrumentos durante a visita domiciliar, sendo registradas em formulário estruturado contendo perguntas fechadas e itens de observação clínica. Todos os instrumentos utilizados possuem validação prévia na literatura. No caso dos DEs da NANDA-I, a validade decorre da padronização internacional, enquanto o Índice de Katz e a Escala de Braden apresentam evidências consolidadas de uso clínico.

### Análise dos resultados e estatística

Realizou-se inferência diagnóstica por duas pesquisadoras a partir da ocorrência dos indicadores clínicos dos DEs na amostra. Quando não houve consenso, uma terceira pesquisadora foi acionada para decisão final.

Os dados foram organizados por meio do programa *Microsoft Office Excel^®^,* e a análise descritiva das variáveis foi realizada através do programa estatístico *Statistical Package for the Social Sciences*, versão 25.0. Foram realizadas estatísticas descritivas por meio de frequências relativas e absolutas, além de medidas de tendência central e dispersão. Ainda foi utilizado o teste de Kolmogorov-Smirnov para a averiguação da normalidade dos dados.

As medidas inferenciais foram obtidas por meio do teste qui-quadrado de Pearson, e, quando as frequências esperadas foram menores que cinco, utilizou-se o teste de Fisher. Em todos os testes, adotou-se um nível de significância de 5% (p≤0,05).

## RESULTADOS

A amostra da pesquisa foi constituída por 63 pessoas idosas acamadas atendidas pela APS. Além disso, 57,1% eram do sexo feminino e apresentaram uma média de idade de 83,32 anos (±9,381), com idade mínima de 60 anos e máxima de 98 anos. Referente à faixa etária, identificaram-se as faixas de 60 a 70 anos (9,5%), de 71 a 80 anos (31,7%), de 81 a 90 anos (33,3%), e acima de 90 anos (25,4%).

Com relação ao estado civil, 49,2% eram viúvos. Sobre a raça, a maioria era branca (58,7%), e a minoria, negra (4,8%). De acordo com o grau de escolaridade, 58,8% não eram alfabetizados. Em termos de renda familiar, 77,7% tinham renda de até dois salários mínimos. E no tocante ao número de dependentes, três foi o número mediano. Ademais, 63,5% moravam com os filhos, e 84,1% tinham o familiar como cuidador.

Em relação aos dados clínicos, a maioria das pessoas idosas possui como doenças prévias hipertensão arterial sistêmica (47,6%) e doenças no sistema nervoso central (34,9%), e 11,1% não dispõem de nenhum diagnóstico. Com relação aos medicamentos, destaca-se o uso de anti-hipertensivos (38,1%), antipsicóticos (23,8%) e inibidores acetilcolinesterase (20,6%).

A respeito do tempo de acamado, 41,3% estavam acamados por pelo menos 24 meses, com mediana de 36,6 meses, tempo mínimo de quatro meses e máximo de 912 meses. Entre os motivos que levaram as pessoas idosas à condição de acamados, destacam-se os acidentes e fraturas (22,2%), sequelas de acidente vascular encefálico (19%), Alzheimer (17,4%), e cansaço e fraqueza muscular (15,9%).

Os resultados do teste de Katz, que avalia o nível de dependência para realizar as atividades básicas de vida diária, mostraram que 60% das pessoas idosas acamadas são dependentes para todas as atividades, e 20% dependem de cinco atividades.

O DE RLPPA foi identificado em 100% dos pesquisados. Os fatores de risco (externos) mais relevantes foram atrito em superfície (100%), pressão sobre saliência óssea (100%), forças de cisalhamento (74,6%), uso de lençóis com propriedades de absorção e eliminação insuficientes (68,3%), carga mecânica contínua (71,4%), acesso inadequado a equipamentos apropriados (65,1%), e conhecimento inadequado do cuidador sobre estratégias de prevenção de LPP (63,5%). No tocante aos fatores de risco (internos), destacam-se a atividade física diminuída (100%), conhecimento inadequado sobre estratégias de prevenção de LPP (96,8%), mobilidade física diminuída (93,7%), adesão inadequada ao plano de prevenção de LPP (60,3%) e desidratação (49,2%).

No tocante aos resultados da Escala de Braden, 68,2% das pessoas idosas apresentaram risco de baixo a moderado. Sobre a população em risco, 100% eram pessoas idosas com deficiência física; 96,8% eram indivíduos recebendo cuidados domiciliares; e 31,7% tinham histórico de LPP. Sobre as condições associadas, 38,1% possuíam perfusão tecidual diminuída; 34,9% possuíam condições neurológicas crônicas; 28,6% possuíam doenças no sistema nervoso central; e 27% possuíam circulação prejudicada.

As características definidoras do DE LPPA indicaram a prevalência de 25,4% do diagnóstico na população de pessoas idosas acamadas assistidas pela APS. Além disso, os participantes da pesquisa apresentaram as seguintes características: área arroxeada em pele intacta e descorada (17,5%); eritema (14,3%); perda de espessura parcial da derme (14,3%); dor em pontos de pressão (11,1%); perda total da espessura do tecido (6,3%); perda total da espessura do tecido com exposição muscular (1,6%); perda total da espessura do tecido com exposição óssea (1,6%); úlcera coberta por escara (1,6%); e úlcera coberta por tecido necrótico (1,6%).

Perante o exposto, verifica-se que, das pessoas idosas acamadas que apresentaram o DE LPPA, 17,5% desenvolveram apenas uma lesão, 4,8%, duas lesões, 1,6%, cinco lesões, e 1,6%, sete lesões. As classificações dessas lesões variaram entre estágio 1, estágio 2, estágio 3, tissular profunda e não estadiável.

As pessoas idosas com e sem a LPP foram relacionados às seguintes variáveis: caracterização sociodemográfica (idade, sexo, raça, estado civil, escolaridade, fonte de renda individual, renda familiar, número de dependentes, arranjo de moradia e existência de cuidador); dados clínicos gerais (doenças previas, medicação em uso, tempo e motivo de acamamento); caracterização clínica da pele (integridade, coloração, umidade, textura, turgor, espessura, sensibilidade, temperatura, mobilidade e edemas); indicadores clínicos dos DEs de RLPPA e LPPA (fatores de risco, população em risco e condições associadas).

A Tabela 1 apresenta as variáveis sociodemográficas e clínicas, bem como os indicadores de DEs que mostraram associação estatisticamente significativa com a ocorrência de LPP em pessoas idosas acamadas.

**Tabela 1:** Associação estatística significativa entre pessoas idosas acamadas que apresentaram a lesão por pressão em relação às que não possuíam a lesão por pressão, a partir das variáveis sociodemográficas e clínicas e dos indicadores dos diagnósticos de enfermagem Risco de Lesão por Pressão no Adulto e Lesão por Pressão no Adulto, Caicó, Rio Grande do Norte, Brasil, 2023

Variáveis	Valor de *p*
Uso de medicamentos	0,024^†^
Pele não íntegra	<0,001 ^*^
Mancha senil	<0,001 ^*^
Sensibilidade dolorosa	0,021 ^*^
Sensibilidade tátil	0,021 ^*^
Pele rugosa	0,056^†^
Turgor diminuído	0,035 ^*^
Eritema	0,002 ^*^
Umidade excessiva	0,032 ^*^
Uso de lençóis com propriedades de absorção e eliminação da umidade insuficientes	0,056 ^*^
Indivíduos com histórico de lesão por pressão	0,015 ^*^

* *Teste qui-quadrado de Pearson; †Teste exato de Fisher.*

A associação entre as pessoas idosas acamadas com LPP em relação às sem LPP, a partir do nível de significância 5%, evidenciou relevância estatística entre 11 variáveis, que foram interpretadas como os fatores associados à ocorrência da LPP, sendo elas: pele não íntegra; eritema; mancha senil; uso de medicamentos; pele rugosa; turgor diminuído; umidade excessiva; uso de lençóis com propriedades de absorção e eliminações da umidade insuficientes; indivíduos com histórico de LPP; sensibilidade dolorosa; e sensibilidade tátil.

## DISCUSSÃO

A prevenção e o manejo precoce da LPP devem ser realizados de forma integrada, considerando aspectos clínicos, educacionais e gerenciais^(6)^. Este estudo analisou o perfil sociodemográfico e clínico, a ocorrência dos DEs RLPPA e LPPA, bem como os fatores associados ao desenvolvimento da LPP em pessoas idosas acamadas atendidas na APS. Tal enfoque reforça o uso clínico dos DEs como instrumento orientador de tomada de decisão e de gestão do cuidado domiciliar.

O perfil sociodemográfico identificado mostrou predominância do sexo feminino, raça branca, estado civil viúvo e baixa escolaridade, com média de idade de 83,32 anos (±9,381). Tais achados são semelhantes a outras pesquisas em atenção domiciliar^(15)^, confirmando o envelhecimento populacional com destaque para a faixa etária de 80 anos ou mais. Além disso, a maioria possui baixa renda familiar, revelando vulnerabilidade social diante das demandas de cuidado^(15)^.

No âmbito clínico, destacaram-se hipertensão arterial, doenças do sistema nervoso e uso frequente de anti-hipertensivos e antipsicóticos, além de causas de acamamento como acidentes, fraturas, sequelas de acidente vascular cerebral e Alzheimer, em consonância com a literatura^(16)^. O teste de Katz confirmou alta dependência funcional, evidenciando a necessidade de estratégias de apoio ao cuidador, como oficinas educativas, visitas domiciliares preventivas e uso de *checklists* para monitoramento^(17)^. Tais práticas de promoção da saúde são fundamentais para reduzir riscos e fortalecer uma cultura preventiva no domicílio.

As alterações cutâneas relacionadas ao envelhecimento, como diminuição da elasticidade, mobilidade aumentada, textura fina e presença de manchas senis, aumentam a vulnerabilidade da pessoa idosa acamada à LPP^(18,19)^. O DE RLPPA foi identificado em 100% da amostra, reforçando a prevalência de risco nessa população^(20)^. A identificação precoce pelo enfermeiro é essencial para prevenir complicações, o que evidencia a importância do PE^(8)^. Esses resultados dialogam com políticas públicas vigentes, como a Política Nacional de Saúde da Pessoa Idosa e as recomendações da Agência Nacional de Vigilância Sanitária (ANVISA) sobre segurança do paciente^(7,21)^, reforçando a LPP como indicador estratégico de qualidade da atenção e da necessidade de alinhamento com protocolos nacionais de cuidado.

A prevalência de 25,4% do DE LPPA encontrada neste estudo é semelhante à de outras pesquisas, que apontam taxas entre 0,5% e 22,5%^(21-23)^. Esse dado reforça a LPP como sério problema de saúde pública no âmbito domiciliar e desafio para a prática clínica da enfermagem. A institucionalização de protocolos de triagem, monitoramento cutâneo e fluxos de cuidado podem potencializar a prevenção e qualificação da assistência, além de se articular com políticas nacionais de atenção à saúde do idoso e de segurança do paciente.

Entre os fatores significativamente associados à LPP, destacaram-se pele não íntegra (p<0,001), mancha senil (p<0,001) e eritema (p=0,002), alterações diretamente observáveis no exame clínico e que configuram sinais de risco elevado. Variáveis como uso de medicamentos (p=0,024), turgor diminuído (p=0,035), umidade excessiva (p=0,032), histórico prévio de LPP (p=0,015) e alterações de sensibilidade dolorosa e tátil (p=0,021) também mostraram relevância, corroborando achados de estudos anteriores^(13,24-30)^. Esses resultados indicam pontos críticos que devem ser priorizados em protocolos de prevenção e monitoramento sistemático.

O uso de medicações contínuas, como anti-hipertensivos, antipsicóticos e sedativos, foi associado a uma maior vulnerabilidade, pois reduzem a perfusão tecidual, comprometem o nível de consciência e prejudicam a mobilidade, dificultando a mudança de posição espontânea^(18,25,31,32)^. Doenças neurológicas, como acidente vascular cerebral e Alzheimer, também interferem na percepção sensorial, aumentando o risco de LPP^(26,27)^. Além disso, histórico prévio de LPP eleva a probabilidade de recorrência, tornando a prevenção ainda mais desafiadora^(33)^.

Os achados deste estudo não apenas revelam vulnerabilidades clínicas, mas também fornecem perspectivas práticas para a APS, como implementação de protocolos de triagem cutânea, fortalecimento da capacitação permanente dos profissionais de enfermagem, uso de instrumentos de apoio ao cuidador e incorporação de tecnologias digitais de monitoramento. Futuras pesquisas devem aprofundar a análise da magnitude das associações encontradas por meio de delineamentos longitudinais e multivariados, bem como avaliar a efetividade de estratégias educativas e interdisciplinares no contexto domiciliar.

### Limitações do estudo

As limitações metodológicas do presente estudo são o delineamento transversal e a ausência de avaliação nutricional precisa, devido à impossibilidade de obtenção do Índice de Massa Corporal. Apesar disso, os resultados obtidos fornecem informações concretas para a qualificação da assistência, com foco na elaboração de protocolos assistenciais que direcionem os cuidados com a pele da pessoa idosa acamada. Estudos futuros, com delineamentos longitudinais e avaliação nutricional sistemática, poderão ampliar a compreensão sobre fatores preditores da LPP, fortalecendo a base científica para protocolos preventivos.

Destaca-se ainda a ausência de análise de medidas de associação, como o *Odds Ratio* e seus Intervalos de Confiança, que poderiam indicar a magnitude do efeito das variáveis associadas à LPP. Dessa forma, os achados apontam a existência de associações estatisticamente significativas, mas não permitem afirmar a força dessas relações. Sugere-se que pesquisas futuras realizem análises multivariadas para aprofundar a compreensão dos fatores predisponentes.

### Contribuições para a área da enfermagem, saúde ou políticas públicas

As contribuições do estudo, a partir da identificação de fatores associados à ocorrência da LPP, reforçam a necessidade de fortalecimento das ações de prevenção da LPP no âmbito da APS. As equipes da ESF devem ser protagonistas na identificação precoce dos fatores de risco e na implementação de estratégias educativas e assistenciais no cuidado domiciliar. O PE e o uso qualificado de DEs, como RLPPA e LPPA, podem orientar condutas baseadas em evidências, promover o monitoramento contínuo da integridade da pele e apoiar decisões clínicas que previnam complicações evitáveis.

Nesse contexto, recomenda-se a criação de instrumentos práticos de apoio à atuação da enfermagem, como *checklists* de risco, oficinas formativas e protocolos interdisciplinares voltados à prevenção da LPP na APS a partir dos resultados do presente estudo. Alinhar essas contribuições às diretrizes da Política Nacional de Saúde da Pessoa Idosa e às recomendações da ANVISA sobre segurança do paciente reforça o papel estratégico da APS na prevenção de agravos evitáveis. Além disso, práticas de promoção da saúde, como educação em autocuidado, capacitação de cuidadores e campanhas comunitárias, podem ampliar o alcance preventivo para além do domicílio. Sugere-se que futuras investigações explorem a efetividade de protocolos interdisciplinares e de tecnologias digitais de monitoramento cutâneo no contexto domiciliar, ampliando o potencial de aplicação prática dos achados.

## CONCLUSÕES

Foram identificados como fatores associados à ocorrência da LPP em pessoas idosas acamadas na APS, como pele não íntegra, eritema, mancha senil, uso de medicamentos, pele rugosa, turgor diminuído, umidade excessiva, uso de lençóis com absorção insuficiente, histórico prévio de LPP e redução das sensibilidades dolorosa e tátil. Esses achados apontam vulnerabilidades clínicas que, se reconhecidas precocemente, podem subsidiar intervenções estratégicas de prevenção no território.

A ocorrência dos DEs RLPPA em 100% da amostra e LPPA em 25,4% evidencia a alta exposição e o impacto real do agravo nessa população. Esses resultados podem subsidiar a elaboração de protocolos municipais de prevenção, integrando o monitoramento cutâneo às visitas domiciliares da ESF e contemplando planos de cuidado personalizados, educação permanente das equipes de enfermagem, capacitação de cuidadores, monitoramento sistemático da pele, intervenções precoces e estratégias de controle ambiental. A incorporação de ferramentas digitais de apoio à decisão clínica e de aplicativos educativos voltados aos cuidadores representa uma inovação promissora para ampliar a efetividade dessas estratégias.

Nesse sentido, o estudo fornece subsídios para fortalecer o papel central dos profissionais da ESF, especialmente enfermeiros, na identificação precoce, prevenção e mitigação da LPP, utilizando o PE como ferramenta norteadora. Recomenda-se a realização de estudos longitudinais e multicêntricos para avaliar a progressão dos fatores identificados e ensaios de intervenção voltados à capacitação de cuidadores e equipes de enfermagem. Espera-se, assim, contribuir para o fortalecimento das práticas assistenciais e para a preservação da integridade cutânea, dignidade e qualidade de vida da pessoa idosa no domicílio.

## Data Availability

Os dados de pesquisa estão disponíveis no corpo do artigo.
